# Fluvastatin protects cochleae from damage by high-level noise

**DOI:** 10.1038/s41598-018-21336-7

**Published:** 2018-02-14

**Authors:** Claus-Peter Richter, Hunter Young, Sonja V. Richter, Virginia Smith-Bronstein, Stuart R. Stock, Xianghui Xiao, Carmen Soriano, Donna S. Whitlon

**Affiliations:** 1 Department of Otolaryngology, Northwestern University Feinberg School of Medicine, Chicago, United States of America; 2 Department of Cell and Molecular Biology, Northwestern University Feinberg School of Medicine, Chicago, IL United States of America; 3 Advanced Photon Source, Argonne National Laboratory, Argonne, United States of America; 4 Department of Biomedical Engineering, Northwestern University, Evanston, United States of America; 5 The Hugh Knowles Center, Department of Communication Sciences and Disorders, Northwestern University, Evanston, United States of America; 6 Interdepartmental Neurosciences Program, Northwestern University, Chicago, United States of America

## Abstract

Exposure to noise and ototoxic drugs are responsible for much of the debilitating hearing loss experienced by about 350 million people worldwide. Beyond hearing aids and cochlear implants, there have been no other FDA approved drug interventions established in the clinic that would either protect or reverse the effects of hearing loss. Using Auditory Brainstem Responses (ABR) in a guinea pig model, we demonstrate that fluvastatin, an inhibitor of HMG-CoA reductase, the rate-limiting enzyme of the mevalonate pathway, protects against loss of cochlear function initiated by high intensity noise. A novel synchrotron radiation based X-ray tomographic method that imaged soft tissues at micrometer resolution in unsectioned cochleae, allowed an efficient, qualitative evaluation of the three-dimensional internal structure of the intact organ. For quantitative measures, plastic embedded cochleae were sectioned followed by hair cell counting. Protection in noise-exposed cochleae is associated with retention of inner and outer hair cells. This study demonstrates the potential of HMG-CoA reductase inhibitors, already vetted in human medicine for other purposes, to protect against noise induced hearing loss.

## Introduction

Hearing loss can be caused by exposure to sound levels that damage the inner ear^1–14^, iatrogenic intervention such as treatment with ototoxic drugs^15–18^, or diseases including infections and genetic syndromes^19–26^. The impact of hearing loss on our society is reflected by the fact that one in six Americans exhibits a mild-to-severe hearing loss^27–30^. An associated impact on earning power as well as on societal integration is documented by a variety of review papers^27–30^. Despite the apparent problem, there are no drugs that are FDA approved to protect, rescue, or repair cochlear damage^29^. Today, treatment of hearing loss is typically limited to amplification of the sound signal (hearing aids) or the surgical implantation of cochlear implants in severe-to-profound deafness. Systemic or local application of drugs has been used with only limited success^29,31^. Emerging approaches to treatment methods include gene manipulation and stem cell therapy^31^, but none are yet viable for human hearing medicine.

In the cochlea of a normal hearing subject, soft tissue structures transform acoustically induced vibrations into trains of action potentials, which are carried to the brain where they are perceived as sound^32–35^. Outer and inner hair cells play a key role in this mechano-electrical transduction^35–41^. Hair cells are the primary receptor cells in the cochlea and are connected via bipolar spiral ganglion neurons to the brain stem^42,43^. Noise exposure, depending on intensity and duration, can cause damage or loss of the hair cells, of the neuron-hair cell synapses, and/or degeneration of neurons, all of which have the potential to cause hearing impairment^11,44^. Since neither hair cells nor spiral ganglion neurons and their synapses are capable of regenerating spontaneously, the damage is permanent.

We recently discovered, using an *in vitro* small molecule screen, that statins, inhibitors of HMG-CoA reductase, increase the neurite length of cultured spiral ganglion neurons^45^. To begin the process of translating the findings from the dish to the clinic, we evaluated the effect of fluvastatin on noise induced hearing loss in guinea pigs *in vivo*. Auditory Brainstem Responses (ABRs) were used to demonstrate that fluvastatin, when present one week before, or after noise insult, and especially at the same time or shortly (<24 h) after noise insult, protects against noise induced hearing loss. Reconstructed images from a novel coherent hard X-ray scanning protocol allowed qualitative analyses of internal cochlear soft tissue structures. A quantitative approach using classical histology indicates that hair cells are protected from degeneration in noise exposed cochleae treated with fluvastatin.

## Results

### Fluvastatin, pumped into the left cochlea, prevents permanent threshold elevation in the right cochlea after noise trauma

To demonstrate that fluvastatin protects the ear from noise-induced damage, a cannula (~180 µm in diameter) connected to an ALZET mini-osmotic pump (model 2004, DURECT Corporation, Cupertino, CA), was implanted in the guinea pig’s *left* cochlea. The pump delivered fluvastatin (50 µM) in DMSO (0.5%) and saline solution at 0.25 µl/h for 28 days. All fluvastatin treated animals were exposed to noise (4–8 kHz, 120 dB re 20 µPa, for 4 h). Four experimental groups were compared: (1) the “fluvastatin before noise” group of animals was implanted seven days before noise exposure, (2) the “fluvastatin at noise” group of animals was implanted directly before the noise exposure, (3) the “DMSO at noise” group of animals was implanted directly before the noise exposure and received DMSO but no fluvastatin, and (4) in the “fluvastatin after noise” group the fluvastatin injection started seven days after noise exposure. One group of animals (“no procedure”) did not receive fluvastatin (no implantation, no noise exposure). The “noise only” group was exposed to noise but received no other treatment.

**Figure 1:**
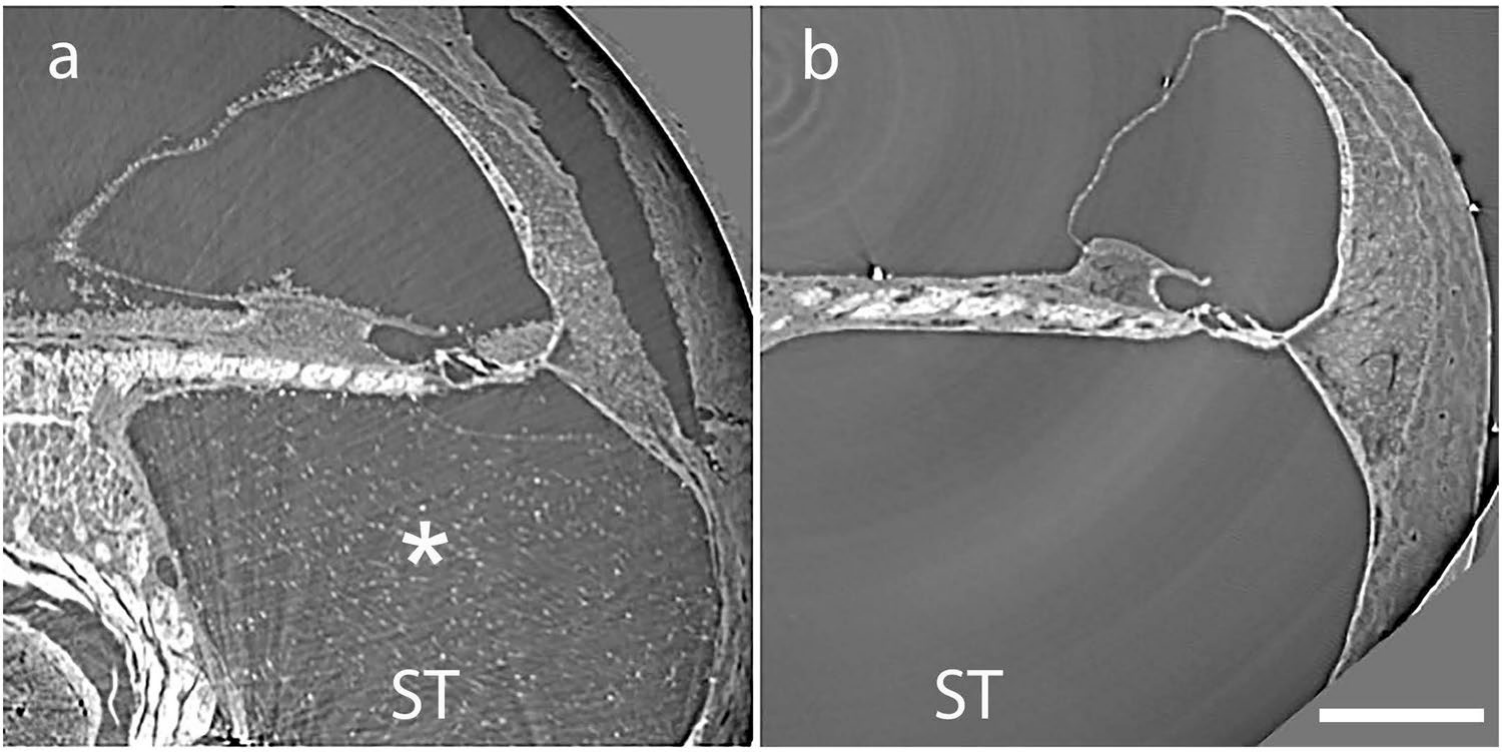
Tissue growth affects cochlear function in the implanted ear. Cross sectional micro-CT images of the cochlear basal turn. The figure shows tissue growth (white asterisk) in scala tympani (ST) of an implanted cochlea (**a**) but not in the basal turn of the non-implanted one (**b**).

In our earliest experiments, we discovered that the surgical insertion of a cannula for delivery of the drug to scala tympani of the guinea pig’s basal cochlear turn, elicited a fibrocytic biological response (micro-computed tomography (micro-CT) images, Fig. 1a, asterisk), which was not seen in any of the non-implanted cochleae (Fig. 1b). We were concerned that this tissue growth together with the surgical procedure might interfere with the cochlear micromechanics, the delivery of the drug, the response to noise stimuli and alter the threshold for ABRs in the left ear. Acoustic click evoked ABR threshold elevations in the *left* ear at the end of the experiment (as compared to the baseline, before noise threshold) are presented in the online supplemental material (SFigure 1). The expected elevation in threshold after exposure to high level noise with (40.7 ± 27.1 dB) or without (30.0 ± 21.4 dB) the surgical insertion of a cannula containing the DMSO/saline carrier is demonstrated. Also expected was the negligible change in threshold in the untreated, “no procedure” group (4.2 ± 4.9 dB). In fact, whenever the cannula with fluvastatin was surgically inserted into the *left* cochlea of noise exposed animals average ABR threshold changes were elevated. Elevations were 43.6 ± 14.9 dB, 25.0 ± 16.5 dB, 49.2 ± 18.8 dB for fluvastatin treatment starting 7 days before, at and 7 days after noise exposure respectively. All differences relative to the “no procedure” group were statistically significant (ANOVA followed by the Tukey Test; DF = 45, F = 4.64, Fc = 2.45). These data initially caused us to question the effect of fluvastatin on noise induced hearing loss in the left ear.

**Figure 2:**
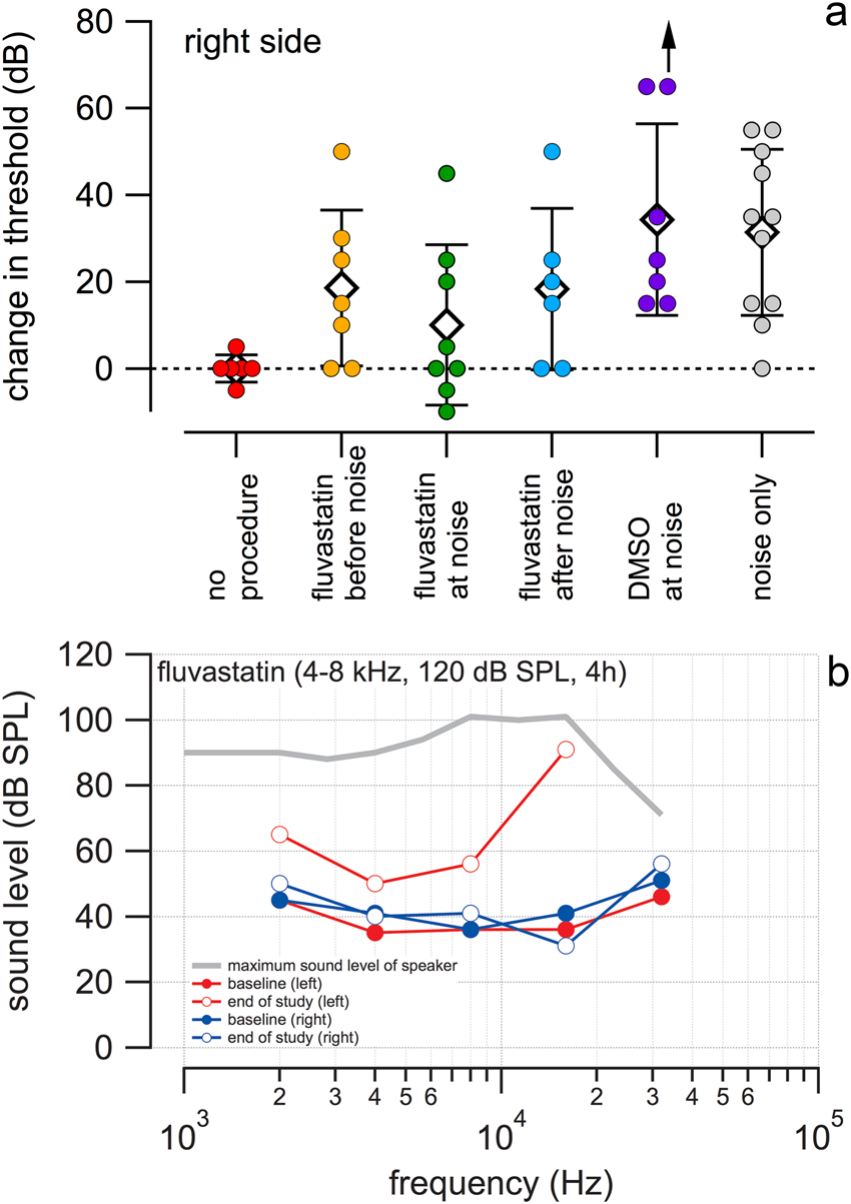
Fluvastatin protects cochlear function. Responses to acoustic click stimuli delivered to the right ear are shown in (**a**). Average threshold elevations (average ± 1 standard deviation) for treatments with fluvastatin before, at, and after the noise exposure were 18.6 ± 18.0 (N = 7), 10.0 ± 18.5 (N = 8), 18.3 ± 18.6 dB (N = 6), respectively. DMSO only treated animals had an average threshold elevation of 34.3 ± 19.1 dB (N = 7). (Note that one ear did not respond to noise at even at the highest level (upward arrow). That ear was calculated as if it responded to the highest level noise, making its calculated threshold shift artificially low (see methods)) For non-treated (no surgery) but noise exposed animals the threshold elevation was 31.4 ± 19.1 dB (N = 11); for animals without any procedure the average threshold change was 0 ± 3.2 dB (N = 6). By ANOVA, differences are statistical significant for the following pairs: no procedure-fluvastatin after noise, no procedure-noise only, fluvastatin before noise-fluvastatin after noise, fluvastatin before noise-noise only, fluvastatin at noise-fluvastatin after noise only, and fluvastatin at noise-noise only. (**b**) Auditory brainstem response (ABR) thresholds obtained from one noise exposed, animal with fluvastatin administered to the left cochlea at the time of exposure. Pure tone ABR thresholds were determined for the left (red traces) and right cochleae (blue traces). Baseline ABR thresholds of this animal at the initiation of the study were similar to our composite no procedure group (gray broken line (**a**)). At the end of the study, ABR thresholds were not elevated for the right cochlea but only for the left, implanted cochlea (**b**).

However, at the same time, we found unexpectedly that the *right* and surgically unaltered cochleae with no fibrocytic reaction was responding to the fluvastatin pumped into the *left* ear with lower threshold shifts after noise exposure. Figure 2a shows the effects in the right cochlea of noise-exposed animals for fluvastatin delivered in left side. Changes in acoustic click driven ABR thresholds (difference between the end of study threshold and the pre-noise exposure baseline) for each animal are plotted. Average threshold elevations in the right cochlea for treatments with fluvastatin before, at, and after the noise exposure were 18.6 ± 18.0, 10.0 ± 18.5, 18.3 ± 18.6 dB, respectively and 31.4 ± 19.1 dB for non-treated (no surgery) but noise exposed animals (Fig. 2a). The average threshold change in animals without any procedure was 0 ± 3.2 dB and for DMSO only treated animals 34.3 ± 19.1 dB. Differences are statistically significant if animals exposed to no procedures or animals treated with fluvastatin at the noise exposure are compared with noise only exposed untreated animals (ANOVA followed by the Tukey Test; DF = 44, F = 3.74 Fc = 2.46). The results show that fluvastatin protects against noise induced ABR threshold shifts when present at the same time (within 24 hours) of noise exposure.

A side-by-side comparison of the data from both ears (SFigure 1) revealed that the threshold shifts were similar when no surgery or surgery with just DMSO was performed (“no procedure” and “noise only’ groups”). In the *left* cochleae, groups that underwent a surgical procedure with fluvastatin showed an approximately 20 dB larger threshold elevation than right cochleae from the same groups of animals. Interestingly, even on the *left* side, the average threshold elevations were less for the animals that received fluvastatin at the time of noise exposure than for the noise only or DMSO groups. The finding that the right cochlea responded to fluvastatin delivered to the left cochlea was advantageous. With this knowledge, we were able to quantify effects of fluvastatin administration on noise induced hearing loss using a pristine, unoperated right ear, lacking the cannula and its associated biological effects including the aberrant tissue growth.

Click responses only provide a gross evaluation of cochlear function. Pure tone thresholds, however, can provide information on the effects of noise and drugs on specific regions of the frequency mapped cochlear spiral. To determine which site along the cochlear spiral was most affected by the noise exposure, we also monitored cochlear function with acoustic tone bursts of different frequencies -, 2, 4, 8, 16 and 32 kHz. In the example in Fig. 2b, threshold sound levels to evoke an auditory brainstem responses (ABR) in one guinea pig were determined for each frequency. The solid circles show baseline ABR thresholds (before exposure) for the right (blue) and left (red) ears (red solid circles). After implantation on the left side and after noise exposure (both ears), the ABR thresholds obtained from the left ear were elevated at the end of the study (red open circles, Fig. 2b), especially at 10 kHz and higher, while the thresholds obtained from the right cochlea remained unchanged (blue open circles, Fig. 2b). The results show that fluvastatin had minimal effect on the combined damage from surgery and noise expose in the left cochlea, but protected from the noise induced damage in the right cochlea.

**Figure 3:**
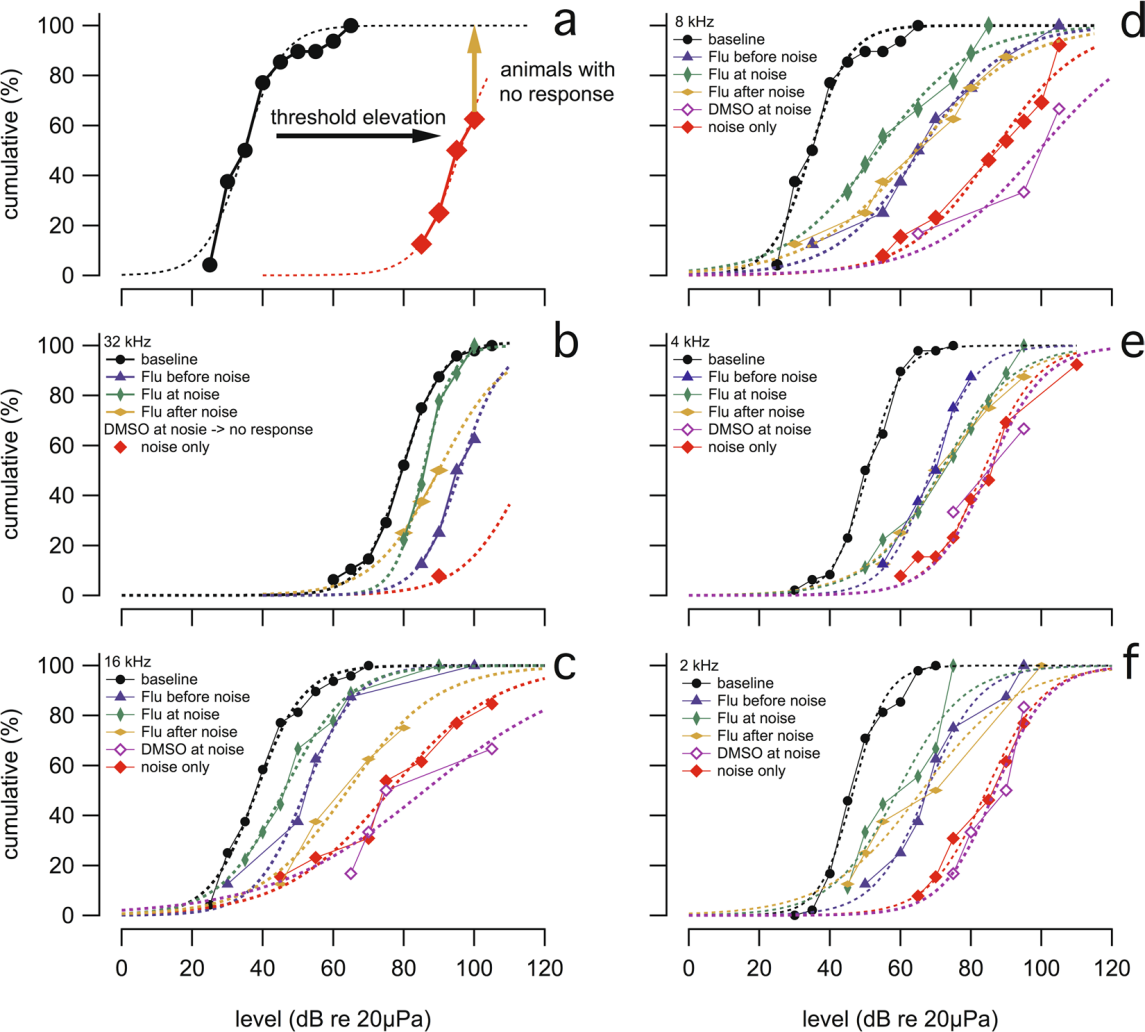
Fluvastatin protects from noise induced hearing loss. The figure shows the cumulative percent histograms of thresholds of the right ears for an auditory brainstem response (ABR) from different animals for various experimental conditions to visualize the distribution of responses in each group: baseline, fluvastatin before noise exposure, fluvastatin at noise exposure, fluvastatin after noise exposure and noise only. Panel (a) provides a key of how to read the results. Shift of the curve towards the right indicates a higher elevation of the threshold to evoke an ABR. The difference between the last data point and the 100%-line on the y-axis provides the percentage of animals that did not have a response to acoustic stimulation up to the highest sound level. The panels (b–f) show thresholds at different stimulus frequencies. The results show a clear elevation in threshold following noise exposure. The injection of fluvastatin into the left cochlea reduces the noise induced threshold elevations, as indicated by the data that graphs to the left of the noise alone data.

Guinea pig stocks are genetically outbred. Each animal in an outbred stock is bred to be genetically unique, and therefore would be expected to show variations in degree of responses to fluvastatin. To get a clearer view of the population distribution of responses to fluvastatin, we plotted cumulative percent histograms of the sound levels required to evoke an ABR (threshold) for various experimental conditions at different frequencies. The histograms in Fig. 3 demonstrate more clearly the benefits of fluvastatin treatment. As indicated in Fig. 3a, a rightward position of a graph indicates an elevation of threshold compared to graphs that are leftward. In Fig. 3b–f, cumulative percent histograms of ABR thresholds are plotted for baseline, fluvastatin before noise exposure, fluvastatin at noise exposure, fluvastatin after noise exposure, DMSO but no fluvastatin at noise exposure and noise exposure only. According to Fig. 3b–f, noise exposure substantially elevates the thresholds of the population. Traces are shifted by about 60 dB to the right side, from the black (baseline) to the red ones (noise only). The difference between the last data point and the 100%-line on the y-axis provides the percentage of animals that did not show a response to acoustic stimulation up to the highest sound level (Fig. 3a). For example at 32 kHz (Fig. 3b), about 90% of the “noise only” animals tested (red marker) did not have a response up to the sound level tested, 105 dB (re 20 µPa). Fluvastatin reduces the noise induced threshold elevations (Fig. 3b–f). The individual pure tone ABR threshold data, their averages and standard deviations are also depicted in SFigure 2. At all frequencies, fluvastatin treated animals demonstrated smaller ABR threshold elevations than animals without fluvastatin. This is particularly apparent at 32 kHz (at the basal region of the cochlea), where often the noise exposure damaged the cochlea enough to prevent measurable ABR threshold responses in the noise only animals. But in fluvastatin treated animals, although elevated, ABR threshold responses could be detected more often (Fig. 3b). Differences between the curves were tested for significance using the Kolmogorov-Smirnov test, which is a nonparametric test to compare a sample with a reference distribution. The data are shown in the traces of Fig. 3. A detailed listing of the outcomes of the statistical testing is given in STable 1 in supplemental materials.

### Inner and outer hair cells are protected by fluvastatin

**Figure 4:**
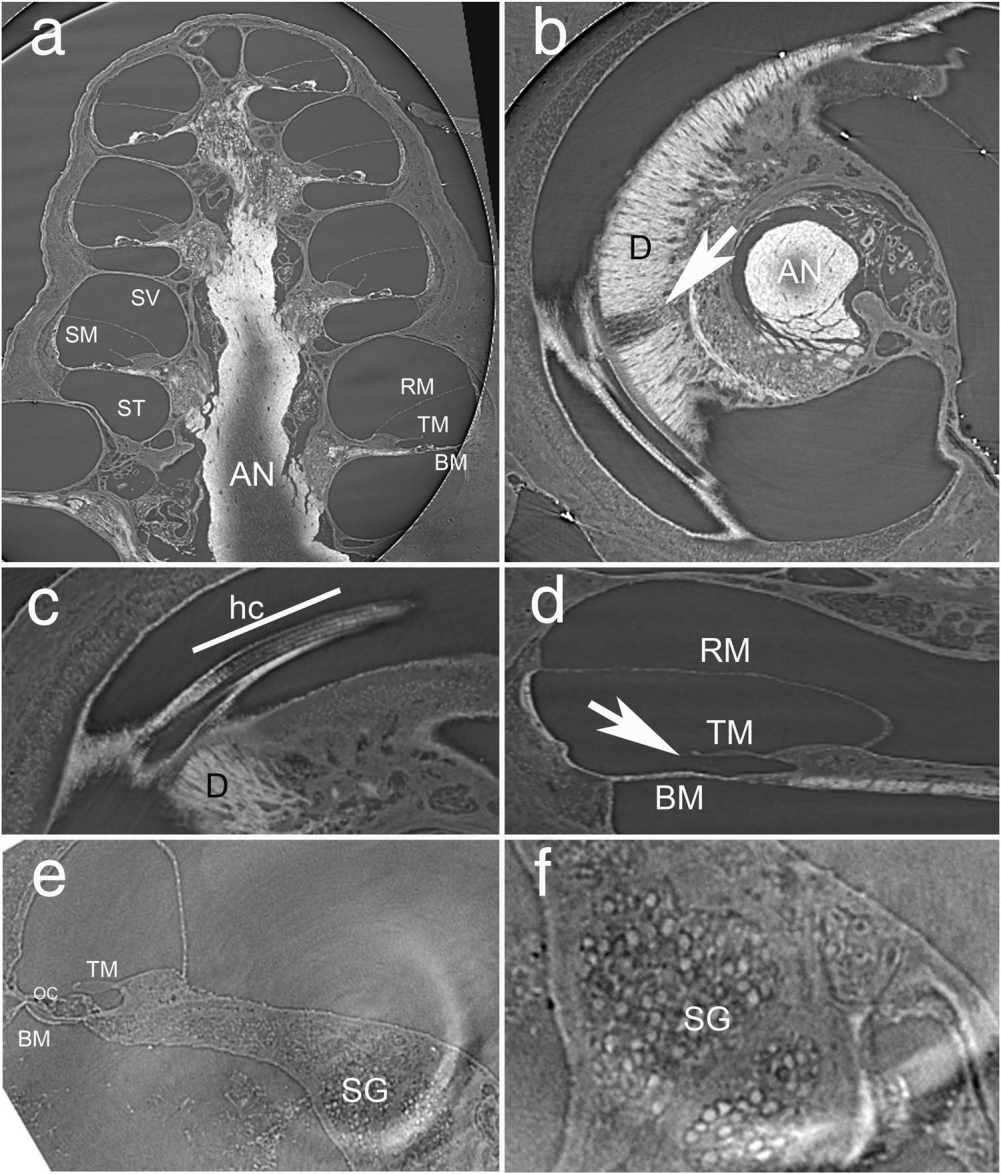
High resolution X-ray micro-CT of the guinea pig cochlea. Tomographic reconstructions of the cochlea allow a free selection of the plane of view. In (**a**) the mid modiolar section of a guinea pig cochlea is shown. In addition to the cochlear wall and the modiolus containing the auditory nerve (AN), scala tympani (ST), scala media (SM) and scala vestibuli (SV), Reissner’s membrane (RM), tectorial membrane (TM), and basilar membrane (BM) can be identified. If the same stack of reconstructed imaged is used, the plane of view can be changed so that it is perpendicular to the modiolus (**b**). Since the cochlea was post fixed with osmium tetroxide, myelinated nerve fibers (D) appear bright in the image. The cochlea was damaged by exposure to high-level noise resulting in a loss of myelinated fibers (arrow). At a different orientation of the section through the stack of reconstructed micro-CT images the hair cells (HC) can be seen, three rows of outer hair cells and the inner hair cells (**c**). Scrolling through the sections it is possible to determine sections in which hair cell loss occurred. A corresponding mid modiolar section (**d**) shows Reissner’s membrane (RM), tectorial membrane (TM) and basilar membrane (BM). The displayed section lacks the organ of Corti (arrow). Panels (e) and (f) show mid modiolar sections of a different, non-exposed cochlea. The intact organ of Corti (OC) is shown in (**e,f**). Spiral ganglion neurons (SG) can be determined in Rosenthal’s canal. Note, the cochlea shown in panels (e) and (f) was not treated with osmium tetroxide; soft tissue structures can be visualized with hard x-rays. The resolution was determined from the images using a high contrast structure such as a bony edge. The spatial resolution is the distance over which the intensity profile drops from 90% to 10% of the initial intensity. It was ~4.5 µm.

To evaluate the anatomical consequences of fluvastatin protection, noise exposed fluvastatin treated and noise exposed untreated cochleae were examined for the presence of inner and outer hair cells using both classical histology and a novel X-ray tomographic approach that images whole, unsectioned cochleae. Virtual sections, which were constructed from the X-ray scans, were used to qualitatively evaluate the cochleae. Locations were identified where missing hair cells or damaged organ of Corti could be visualized. Figure 4 shows examples of virtual sections of whole guinea pig cochleae. All but the hook region are presented. Figure 4a is a mid modiolar plane and shows 3.5 turns of the guinea pig cochlea. The basilar membrane (BM) and its overlying organ of Corti can be identified. Reissner’s membrane (RM) can be seen as a thin line. The auditory nerve (AN) is visible in the center of the modiolus, with slightly less contrast in the lower part of this image due to reduced penetration of the osmium in this cochlea. In a plane selected perpendicular to the modiolus (Fig. 4b,c), the dendrites (D) connecting the neurons with the hair cell in the organ of Corti are visible (Fig. 4b,c). The bright appearance of the myelinated nerve fiber bundles comes from the contrast enhancement with osmium tetroxide. The loss of myelin and corresponding potential loss of nerve fibers can be clearly identified in Fig. 4b (arrow). The distance of this location is 16.2 mm from the apex of the cochlea, which corresponds along the tonotopic map of the cochlea to a best frequency of 26.2 kHz. Using the same stack of reconstructed images a different virtual section can be selected to show the three rows of outer hair cells (hc, Fig. 4c). Zooming in at one turn in a midmodiolar section, one can observe specific loss of cells. For example, in Fig. 4d the basilar (BM), tectorial (TM), and Reissner’s (RM) membranes can be seen, but the entire organ of Corti is missing at this site after noise exposure. The major soft tissue structures of the inner ear can be seen in the cochlea of a non-noise exposed, normal hearing animal (Fig. 4e). The structures include the basilar membrane, the tectorial membrane, the inner and outer pillar cells, and the supporting cells (Fig. 4e). Note, for Fig. 4e,f, no post fixation with osmium tetroxide was conducted. While contrast enhancement with osmium tetroxide can be beneficial in recognizing myelinated structures, it is not required. Spiral ganglion neurons, most myelinated, appear as bright circles (Fig. 4e,f).

**Figure 5:**
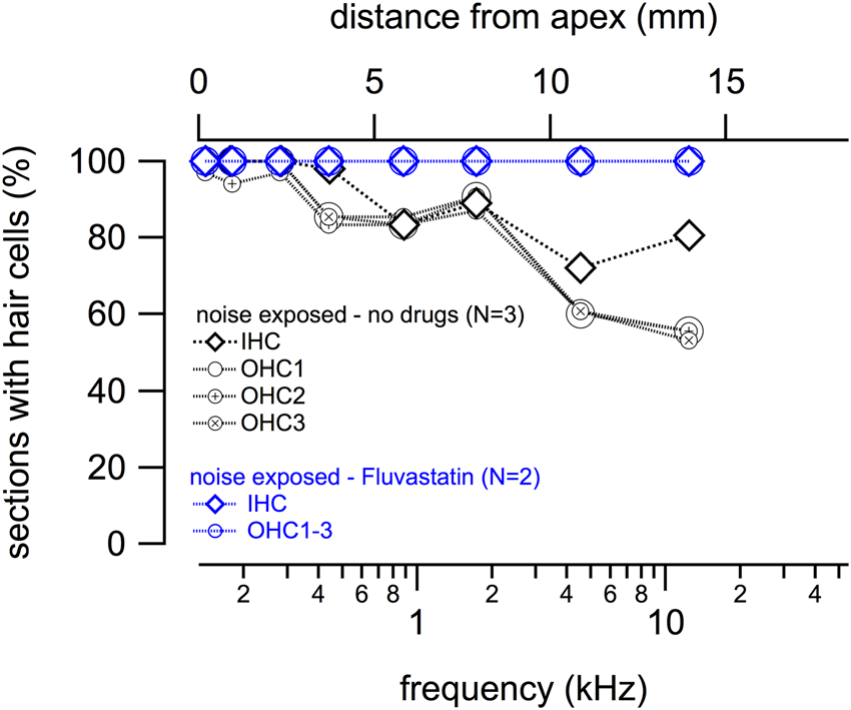
Fluvastatin protects hair cells from degeneration. Right cochleae from animals in the noise only group (noise at 120 dB SPL, 4–8 kHz, 4 h), and fluvastatin treatment at noise exposure group were used to determine hair cell loss. Noise exposed fluvastatin treated cochleae maintained hair cells in every sampled section (**a**). Noise exposed untreated (noise only) animals had hair cell loss most prominent in the cochlear base (**a**). For outer hair cells the loss was observed in up to 50% of sections and for inner hair cells about 20% of sections were missing hair cells.

The X-ray scans allowed the visualization of regions of hair cell loss and nerve fiber loss. The findings were quantified using classical histology. Eighteen consecutive mid-modiolar cochlear sections were examined for the existence of outer and inner hair cells and the probability of finding a hair cell in different regions of each section was calculated (Fig. 5). Noise exposed animals and noise exposed animals treated concurrently with fluvastatin were analyzed. For animals treated with noise alone, hair cells were missing. For animals treated with fluvastatin and exposed to noise, every cut edge of every section had inner and outer hair cells (Fig. 5). For the experimental condition where the fluvastatin is given at noise, the probability of finding a hair cell in the sampled sections was 100%. The probability of finding hair cells in sections from cochleae in the noise exposure group was mapped along different cochlear locations from apex to base. No significant differences in outer hair cell (OHC) or inner hair cell (IHC) probabilities from that in the fluvastatin treated cochleae were observed at the cochlear apex, (tonotopic locations at 2 or 4 kHz; (Fig. 5)). The losses become apparent and increase towards the base (higher frequency regions) of the cochlea. These data indicate that fluvastatin when present within 24 hours of noise exposure protects hair cells from degeneration due to noise.

## Discussion

The impetus for this study came from our recent small molecule screen^45^. The NIH Clinical Collection (a library of small molecules) was screened for the ability to promote neurite elongation from dissociated spiral ganglion neurons. One hit was found: the HMG-CoA reductase inhibitor “cerivastatin”. Further work indicated that most, but not all statins were active in the assay. We did not initially plan to study statins in noise induced hearing loss, but once the statins were implicated in spiral ganglion neurite elongation, we chose fluvastatin, which is still in clinical use and was active in the assay, to elevate to in depth study in the guinea pig model of noise induced hearing loss.

It often happens that compounds identified in small molecule screens never make it to the drug stage due to inactivity, reduced permeability, biochemical alterations or unexpected side effects such as toxicity or off target activities. We accepted this as a possibility in our own experiments as we went forward, initially aiming to demonstrate fluvastatin effects on damaged spiral ganglion neurites in the absence of hair cells. What was not expected was the advantageous “side effects” – protection of ABR threshold and maintenance of hair cells - exerted by delivery of fluvastatin to a noise exposed cochlea and this interfered with our ability to measure neurite regeneration in in the absence of hair cells. Moreover, the effect of fluvastatin delivered to the left ear was exerted in the pristine, un-operated contralateral ear, lacking the cannula and its associated biological effects including the aberrant tissue growth. Using the contralateral ear, this paper focuses on the unexpected “side effects” and demonstrates that fluvastatin protects against noise induced ABR threshold shifts in the guinea pig.

Exposure to high sound levels has a devastating effect, the loss of hearing. Exposure time, frequency spectrum of the noise, and level of the sound determine whether or not damage to the ear occurs^10,11,46^. According to the literature, the most vulnerable cells are the outer hair cells^47–50^. Following noise exposure the three rows of outer hair cells are disarranged, OHC bodies and nuclei as well as mitochondria are swollen, and the stereocilia are damaged. Positive staining with TUNEL or caspase-3 indicates that the noise damage can lead to the activation of the apoptosis pathway. Following the noise insult, OHC degeneration continues and expands for days and weeks^49,51^. The time over which the degeneration of the neural structures occurs differs between animal species and can progress over months or years^52–55^. Increases in oxidative stress^56–59^, in the inflammatory responses^60,61^ and alterations in the cytoskeletal structure, are all part of the known downstream responses of cochleae to noise exposure. In other tissues, it is significant that statins, through their effects on the non-cholesterol branches of the mevalonate pathway, have been reported to interfere with all three of these mechanisms, and therefore fluvastatin has the potential to exert its protective effects on noise induced hearing loss by mechanisms that are independent of fluvastatin effects on cholesterol.

Our experiments showed that fluvastatin, an inhibitor of the mevalonate pathway, protects hair cells. The mevalonate pathway (MVA) plays a key role in multiple cellular processes by synthesizing the building blocks of sterol isoprenoids, such as cholesterol, and non-sterol isoprenoids, such as dolichol, heme-A, isopentenyl tRNA and ubiquinone. It is important for the production of dimethylallyl pyrophosphate (DMAPP) and isopentenyl pyrophosphate (IPP). Both serve as the basis for the biosynthesis of molecules used in protein prenylation, cell membrane maintenance, hormones, protein anchoring, and N-glycosylation. Statins can regulate the production of these downstream metabolites by inhibiting the rate-limiting enzyme of the MVA pathway, HMG-CoA reductase.

Based on the well-known protective effects of statins on the cardiovascular system, Borghi *et al*. hypothesized that inhibiting the reductase with statins might have a positive effect on sudden sensorineural hearing loss (SSHL) by altering the local hemodynamics in the cochlea^62^. However, only a handful of publications have considered statin use for protection or repair of hearing or cochlear cells, and the results vary. Syka and coworkers, in an age related hearing loss study, demonstrated that atorvastatin (10 mg/kg) given to C57BL/6 J mice showed larger amplitudes of distortion product otoacoustic emissions (DPOAE) than did the non-treated control group; suggesting that atorvastatin preserves outer hair cell function^63^. In the same study, Syka and coworkers observed decreased expression of intercellular and vascular adhesion molecules in the aortic wall of atorvastatin-treated animals. They concluded from the results that reducing endothelial inflammatory effects might contribute to the positive effect of atorvastatin on the amplitudes of DPOAE by improving the blood supply to the inner ear^63^. In 2007, Olzowy *et al*. conducted a clinical trial testing the effect of atorvastatin on hearing and tinnitus in humans^64^. In a double-blind study, patients were randomly assigned to treatment with either atorvastatin (40 mg/d orally) or placebo. Over 13 months, no significant differences were found between the treated and untreated (control) groups.

We are unable to compare our high intensity, one octave band noise exposure and fluvastatin treatment in guinea pigs with a study using mice that were pretreated with a different statin, pravastatin (25 mg/kg for 5 days), before exposing them to noise: 3 h at 112-dB sound pressure level (dB SPL, re 20 µPa) of broad-band white noise (1–20 kHz, about 4.5 octaves^65^. The short span of that study (2 weeks) and the fairly low energy spectral density, make the type of damage, TTS or PTS, difficult to assess. Nonetheless, the study does provide a clear demonstration that oral administration of pravastatin to mice causes changes in the biochemistry of cochlear tissue. Pravastatin administration lowers the concentration of GTP-Rac1, which among other activities is known to bind and activate the NADPH oxidase complex, an enzymatic generator of ROS. The effect pravastatin may have on neurites in noise treated cochleae is unknown. In our *in vitro* assay on dissociated spiral ganglion neurons, pravastatin was unable to promote neurite length regeneration, even when assayed at 25 times the lowest active concentration of fluvastatin^45^.

Brand *et al*.^66^ was able to demonstrate the expression of HMG-CoA reductase in the organ of Corti (contains the hair cells and the supporting cells), the spiral ganglion, and the stria vascularis of 5 day old Wistar rats. They also showed *in vitro* in 5-day old cochlear explants that simvastatin protected against gentamicin induced hair cell damage and that exogenous mevalonate, the product of the HMG-CoA reductase, could reverse the simvastatin protection in this postnatal mouse model. On the other hand, Park *et al*.^67^ reported that simvastatin induced cell death in cultured mouse cochlear neuroblasts (VOT-33).

Here we demonstrate that Fluvastatin, a drug currently in use for lowering serum cholesterol, reduces noise-induced changes in cochlear function at least in part by protecting inner and outer hair cells. The effect is unlikely to be mediated by effects on cholesterol, since reduction in cellular cholesterol by other means, reduces the function and survival of hair cells^68,69^.

In auditory research, the cochlea contralateral to the interventional side is often used as a “control”. We expected to do the same. The protection of ABR thresholds on the right side after left ear injection was unexpected. At present, we do not know how fluvastatin exerts protective effects on the contralateral cochlea. The literature makes very rare remarks on the subject of transfer of chemicals or viruses from one ear to the other^70–73^. Several papers report transfer of viruses between cochleae^72^. Multiple authors raise the possibility of transfer via the cerebrospinal fluid (CSF), either through the cochlear aqueduct or thorough the bone marrow space. We also do not rule out blood-borne transfer, but we deem it unlikely due to the low concentration of fluvastatin we deliver to the left ear (50 µM) and its potentially high dilution in the blood. Nor do we rule out an effect of fluvastatin in CSF on neurons in the brain that would then affect responses in the contralateral cochlea. Regardless, the purposeful delivery of a drug to one cochlea in order to study its effects in the contralateral cochlea has not previously been reported.

Histology, which is necessary to show the integrity of the cochlear structures, can take weeks to months to perform. For example, in our classical histologic study the gross morphology of cochlear tissue is evaluated to determine possible changes resulting from high-level noise exposure. The procedures include fixation, decalcification, embedding, sectioning, and staining of the tissue. Next, images of the sections are taken and analyzed. Typically, existence of cochlear structures and the integrity of the organ of Corti are evaluated. In contrast to classical methods, for X-ray computed tomography, the cochleae must only be decalcified. Scanning of the cochlea typically takes 20 minutes, but can also be accomplished in less than five minutes using novel, fast scanning methods. From the initial projections the entire cochlea can be virtually reconstructed, typically resulting in a stack of up to 2048 slices. A stack of images can be used to create any imaginable cut through the cochlea. This method allows selection for consistent evaluation planes through the cochleae.

The concept of using coherent hard X-rays to image soft tissue structures is not novel but has rarely been explored in cochlea^74–78^. Here we demonstrated that micro-CT with synchrotron X-radiation clearly reveals the cellular structures of the cochlea. The isotropic voxel size is 1.45 µm and the spatial resolution is about 4 µm. The advantages of the technique are the time and flexibility of selecting the plane of interest through the cochlea; scanning and reconstruction of the cochlea can be complete in about 1 hour. Since the cochleae can be scanned in buffered saline solutions, sections of interest can be identified in the micro-CT scan and the cochlear tissue can be optically sectioned accordingly for further investigation. Quantitative measures are possible. The technique has been explored for the cochlea but can be used for many other applications as well. Although the use of synchrotron radiation limits the technique to a few dozen facilities in the world, recent development of commercial desktop micro-CT systems employing phase contrast may allow comparable results to be obtained elsewhere^79,80^.

In conclusion: The results obtained with this study demonstrate fluvastatin protection against noise induced hearing loss, which is associated with retention of inner and outer hair cells. The results stem from cochleae that were not surgically manipulated during the experiments. The results highlight statins as a new class of compounds to explore for protection against hearing loss. For a qualitative evaluation of cochleae, synchrotron radiation based X-ray tomographic method was used. Inner ear structures were imaged at micrometer resolution in unsectioned cochleae. The method reduces traditional tissue processing and anatomical analysis times from weeks to hours. Such an imaging approach has the potential to speed up *in vivo* auditory experiments and to contribute significantly to evaluation of drugs for hearing loss.

## Methods

### Ethics Statement

Care and use of animals followed the guidelines in the NIH Guide for the Care and Use of Laboratory Animals. All efforts were made to minimize pain.

### Animals

Outbred Hartley guinea pigs of both sexes (200–500 g) from Kuiper Rabbit Ranch (Gary, IN) were used for experimental procedures. The imaging examples included here used Hartley guinea pigs from Charles River Laboratories (Toronto, Canada). Animals with elevated baseline thresholds were not included. At the end the experiments, animals with fluid or infection in either ear were removed from the study.

### Monitoring cochlear function

Cochlear function was monitored by recording ABRs evoked by acoustic clicks or pure tone bursts. Voltage commands for the acoustic stimuli were generated with custom written software. Clicks were 50 µs pulses, delivered at different amplitudes via the KPCI 3110 I/O board (Keithley Instruments, Cleveland, OH) of the computer; the 10 ms tone bursts included a 1 ms rise/fall time. The carrier frequency of the tone bursts was varied between 2 and 32 kHz, typically at 1 step per octave. Voltage commands were delivered to an audio amplifier (Alesis, RA150, Cumberland, RI) and were used to drive a Beyer 770 Pro headphone (Bayerdynamics, Farmingdale, NY), which was placed directly in front of the outer ear canal. The sound level at opening of the speaker was determined with a 1/8-inch Brüel and Kjær microphone (Nærum, Denmark) and was between 90 and 119 dB SPL for pure tones and had a peak pressure of 120 dB SPL for the clicks.

For ABRs, animals were anesthetized with isoflurane (3% induction in induction chamber, 1.5% maintenance via nose cone) delivered in a 50/50 mixture of oxygen and nitrous oxide. Three hypodermic needles were inserted under the skin, one at the ipsilateral mastoid bone, one at or near the vertex, and one common reference electrode under the skin at the animals back. Responses to acoustic clicks or tone bursts were band pass filtered, 0.3 to 3 kHz, amplified 10,000 times by a preamplifier (ISO80, World Precision Instruments, Sarasota, FL), and were recorded with a personal computer (PC), which was equipped with a KPCI 3110 I/O board. 512 responses to click stimuli and 100 responses to tone bursts were averaged. The ABR threshold was determined visually by the first appearance of a response in the averaged traces. An example of a baseline recording and the measurements at the end of the study are shown in SFigure 4. Sound levels for the ABR thresholds were averaged and compared among the experimental groups. Non-treated and unexposed animals were described by the term “no procedure” and did not undergo surgery or were exposed to noise. Animals described with the term “noise only” were only exposed to noise. Noise exposure was 4 h of band-limited noise (4–8 kHz) at an average sound level of 120 dB SPL (SPL = sound pressure level referenced to 20 µPa).

Baseline cochlear function (by click evoked and pure tone ABR thresholds) was determined before the implantation of the animals and before the noise exposure. After baseline thresholds were determined to be similar among the animals, all animals were randomly assigned either to the group exposed to noise and receiving fluvastatin or to the group exposed to noise alone. The animals were implanted on their left sides. During recovery from surgery, the animals were placed in a shoe box-sized chamber and were exposed to the noise. Animals were recovered from anesthesia and cochlear function was monitored at least at the end of the study.

### Implantation of the mini-osmotic pump

Surgery used strictly aseptic procedures. The animals received a subcutaneous dose of 0.05 mg/kg Buprenex. Anesthesia was induced with 5% of isoflurane while the animal was placed in an induction chamber and was then maintained with 1–3% of isoflurane in oxygen given via a nose cone throughout the surgery. To access the cochlea, an about 2 cm c-shaped incision was made behind the pinna. The muscles attached to the bulla were gently removed by blunt dissection and an approximately 2 × 2 mm bullotomy was created. The basal turn of the cochlea was visible and a cochleostomy was created with an electric drill (OmniDrill35, World Precision Instruments, Sarasota, FL) and a 1 mm burr. A second hole was created in the bulla. A custom-made catheter consisting of a medical grade tube (TYGON^®^, U.S. Plastic Corp.^®^, Lima, OH) and an insertion attached to an ALZET micro-osmotic pump (Model 2004, 0.25 µl/h, 28 days; Durect, Corp. Cupertino, CA) was inserted into scala tympani of the cochlea’s basal turn. The cochleostomy was sealed with the silicone stopper at the catheter and the tubing was secured with dental acrylic at the bulla. The bulla was sealed with the acrylic and the ALZET pump was placed between the shoulder blades. The incision was closed in two layers using 4-0 vicryl and 4-0 ethilon (Ethicon Inc., Sommerville, NJ). Post-surgical pain management was achieved with Buprenex. For the different experimental groups the ALZET pump contained 250 µl of Fluvastatin (50 µM) in Ringer’s Lactated Solution (RLS; Hospira Inc., Lake Forest, IL) with 0.5% DMSO, in RLS +0.5% DMSO, or the catheter was tied off. The pumps were not primed prior to implantation, which indicates that efficient drug delivery was established about 24 h after the implantation and noise exposure. It should be noted that in case the noise exposure occurred one week before the fluvastatin treatment, the drug was delivered for four weeks and the status of the ear was determined five weeks after noise exposure. In case noise exposure occurred at the same day of the implantation, the end of study was four weeks after the noise exposure and after four weeks of drug treatment. For the remaining noise exposed group the exposure occurred one week after the implantation and at the end of the study cochlear function was determined three weeks after noise exposure and four weeks after drug.

### Sample preparation

At the conclusion of the experiments the animals were cardiac perfused with 0.1 M phosphate buffered solution (PBS), pH 7.4 followed by 4% paraformaldehyde in PBS, and the cochleae were harvested and post fixed in paraformaldehyde at room temperature for at least another hour. Next the cochleae were washed with PBS and placed for about three weeks into 10% ethylenediaminetetraacetic acid (EDTA; Sigma-Aldrich, St Louis, MO) In PBS (pH 7.4) for decalcification. Subsequently, the specimens were transferred into PBS and stored in the refrigerator until imaging. At least one day before imaging, some of the cochleae were immersed in PBS containing 1% osmium tetroxide (Electron Microscopy Science, Hatfield, PA) for 60 minutes.

For imaging, the samples were securely placed into small Eppendorf tubes, which were filled with PBS. Samples were fixed in place to ensure that movements would not occur during imaging. Fluids were then degassed for at least 24 hours. At the Advanced Photon Source (APS) at Argonne National Laboratory, the Eppendorf tubes were mounted in the radiation beam path and the samples were scanned. Following X-ray scanning, Kuiper cochleae were prepared for classical histology.

### Image acquisition and tomographic reconstruction

Micro-CT was carried out at the 2-BM-B beamline of the APS. All cochleae were imaged using monochromatic radiation with photon energies of 22 kilo-electron volts (keV). The separation between the detector and the tomography rotation axis was 600 mm for phase contrast. A 5× objective lens was used in the detector system. The resulting field of view for the 5 × objective lens was about 3 × 3 mm^2^. Specimens were placed such that the cochleae were in the visual field at all times.

A series X-ray projections were taken over a range of 180 degrees at increments of 0.12 degrees. The exposure time for a single image was 0.2–0.3 s. At the beginning and end of each image series, flat field images (no object in the beam path) were recorded; after the series, a dark field image (the radiation beam was blocked) was captured. After each set of projections was recorded, the guinea pig cochlea was translated vertically (along the modiolar axis) to cover a new section of the specimen. Two scans were required to include the entire guinea pig cochlea with the 5× lens.

The projections were used to reconstruct the cochlea. Custom written phase retrieval software (Paganin algorithm) for non-interferometric phase imaging with partially coherent X-rays was used^81–83^. Reconstructions were on a 2048 × 2048 grid with custom written software^84^. The reconstructions resulted in 1.45 µm isotropic voxels. The spatial resolution was determined from the response of the system to a sharp discontinuity in the image such as a bony edge. The gray values along a line which was crossing the edge was used for the measurements. The parameter measured was the distance required for the gray values to fall from 90% to 10%. The resulting distance was 4.4 µm, which was considered the spatial resolution.

### Classical histology

Reconstructions from the X-ray scans were compared with results obtained from classical histology. Cochleae were rinsed in phosphate buffered solution three times for 15 minutes each and were dehydrated in a graded 6-step acetone series (25%, 50%, 75%, 90%, 100% and 100%). Samples were embedded in Araldite-Epoxy Resin (Electron Microscopy Science, Hatfield, PA), 7:1 Acetone:Resin, 1:1 Acetone:Resin, 1:7 Acetone:Resin, one time pure Resin. The plastic was cured for 12 hours in an oven at 60 °C.

Sections were cut perpendicular to the modiolus with an ultramicrotome (LKB 8800 Ultrotome III, Stockholm-Bromma, Sweden) at 7 µm and were placed on glass slides. The sections were stained with 0.05% toluidine blue in 1% sodium tetraborate solution (Sigma Aldrich, St Louis, MO). Images of selected sections were captured with a microscope (Leica DMRB, Buffalo Grove, IL) equipped with a Spot Insight Color camera (SPOT^TM^ Imaging Solutions, Sterling Heights, MI) at 5× and 20×. Images were compared to the images obtained with X-rays.

### Computer aided length measurements

The stacks of tomographic reconstructions were used for rendering the pillar heads along the cochlea. A custom written code in MATLAB allowed selecting pillar heads in reconstructed sections. A cursor was placed over the pillar heads and the position was recorded by mouse click. The step size between sections was freely selectable and determined the resolution of the resulting plot. At the end of the procedure, a 3D-plot of the pillar heads along the cochlea was created and was correlated to the frequency place map for the guinea pig cochlea as published^85,86^. In our example we were able to determine the distance of the cochlear damage along the cochlea and correlate that site with a best frequency.

### Hair cell density

To determine the protective effect of fluvastatin on hair cells, each turn visible in a mid modiolar cross section was inspected to determine whether inner and outer hair cells were present. Eighteen successive sections at 7 µm were inspected. The presence of outer and inner hair cells was scored by the presence of a cell nucleus and stereocilia bundle. Since the sections are smaller than the diameter of the hair cells it is possible that a single hair cell is present in several sections and gaps between hair cells can results with no hair cell in the section despite no hair cell loss having occurred. To account for those limitations, the fraction of sections with a hair cell visible was calculated for animals from the noise only and noise at the time of fluvastatin treatment group. This allowed us to estimate the “probability” of finding a hair cell at each frequency. Each visible cut edge from base to apex was examined.

### Data and statistical analysis

ABR thresholds were entered into IgorPro 6 software (WaveMetrics, Lake Oswego, OR) to calculate threshold shift values by subtracting baseline threshold values from end of study threshold values for each treatment. Individual data are shown in the graphs as well as the average values +/− one standard deviation (SD). For cochleae that did not show any response up to the maximum sound level produced by the speaker the threshold shift was calculated as the difference between baseline and the maximum sound level of the speaker. This value, although it underestimates the threshold shift, was included in the calculation of the average and corresponding standard deviation. We are aware that this procedure biases the values shown towards lower threshold shifts. To better represent the animals without any auditory response to acoustic stimuli cumulative plots were used as described below. The average values of the different experimental groups were compared and the differences were tested for significance. For statistics a one-way analysis of variance (ANOVA) was applied followed by the Tukey test which performs multiple comparisons. Statistical testing was performed with α = 0.05 and p ≤ 0.05.

Cumulative plots from the pure tone data were used to show the threshold distributions among the animals in each group. A decrease in the maximum value indicated that some animals did not show any ABR to acoustic stimuli. Shifts of the traces to the right side indicated an increase in the ABR threshold. To determine whether differences in the traces are significant, the traces for each given frequency were compared with the Kologorov Smirnov (KS) test. It is a non-parametric test to compare the distribution of a reference and a sample. The testing was done with an α = 0.05 and p ≤ 0.05.

Qualitative histological examination of the upper base, middle and upper middle regions of the cochleae was performed to determine cellular integrity of the organ of Corti.

### Data availability statement

All data generated or analyzed during this study are included in this published article (and its Supplementary Information files).

### Study Approval

This study was approved by the Animal Care and Use Committee of Northwestern University and by the Navy Bureau of Medicine and Surgery (BUMED).

## Electronic supplementary material

Supplementary Information
