# Supplementary material for: Fluvastatin protects cochleae from damage by high-level noise
Source: Sci Rep. 2018 Feb 14;8:3033. doi: 10.1038/s41598-018-21336-7 (PMC5813011; doi:10.1038/s41598-018-21336-7)
Supplement: Supplementary file 1 — Supplementary Information [file 41598_2018_21336_MOESM1_ESM.pdf]

Supplementary Information
Fluvastatin protects cochleae from damage by high-level noise

Claus-Peter Richter,[1,4,5] Hunter Young,[1] Sonja V. Richter,[1] Virginia Smith-Bronstein,[1] Stuart R. Stock,[2] Xianghui Xiao,[3] Carmen Soriano,[3] and Donna S. Whitlon[1,5,6]

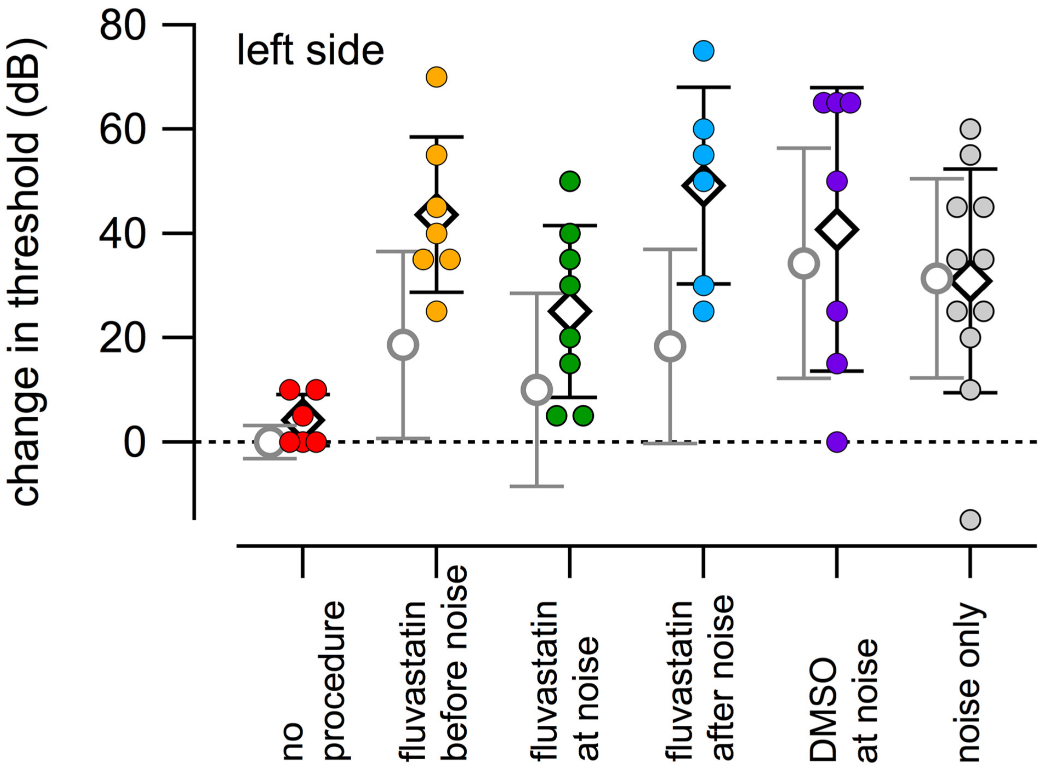

SFigure 1. Cochlear function in the implanted ear
Responses to acoustic click stimuli delivered to the left ear are shown. Average threshold elevations (average ± 1 standard deviation) for treatments with fluvastatin before, at, and after the noise exposure were 43.6 ± 14.9 dB (N=7), 25.0 ± 16.5 dB (N=8), 49.2 ± 18.8 (N=6), respectively. DMSO only treated animals had an average threshold elevation of 40.7 ± 27.1 dB (N=7). For non-treated (no surgery) but noise exposed animals the threshold elevation was 30.0 ± 21.4 (N=11); for animals without any procedure the average threshold change was 4.2 ± 4.9 dB (N=6). Differences were statistically only significant when compared with the untreated animals (DF=39, Fc=2.64, $P<0.05$). The gray circles show the corresponding data obtained in the right ear shown in Figure 2a. The threshold shifts in the left cochlea for the no procedure-unexposed and the noise only conditions were similar to those in the right ears. The other fluvastatin groups showed about a 20 dB higher threshold change in the left cochlea than in the right. The data suggest that surgery introduces a 20 dB elevation in threshold.

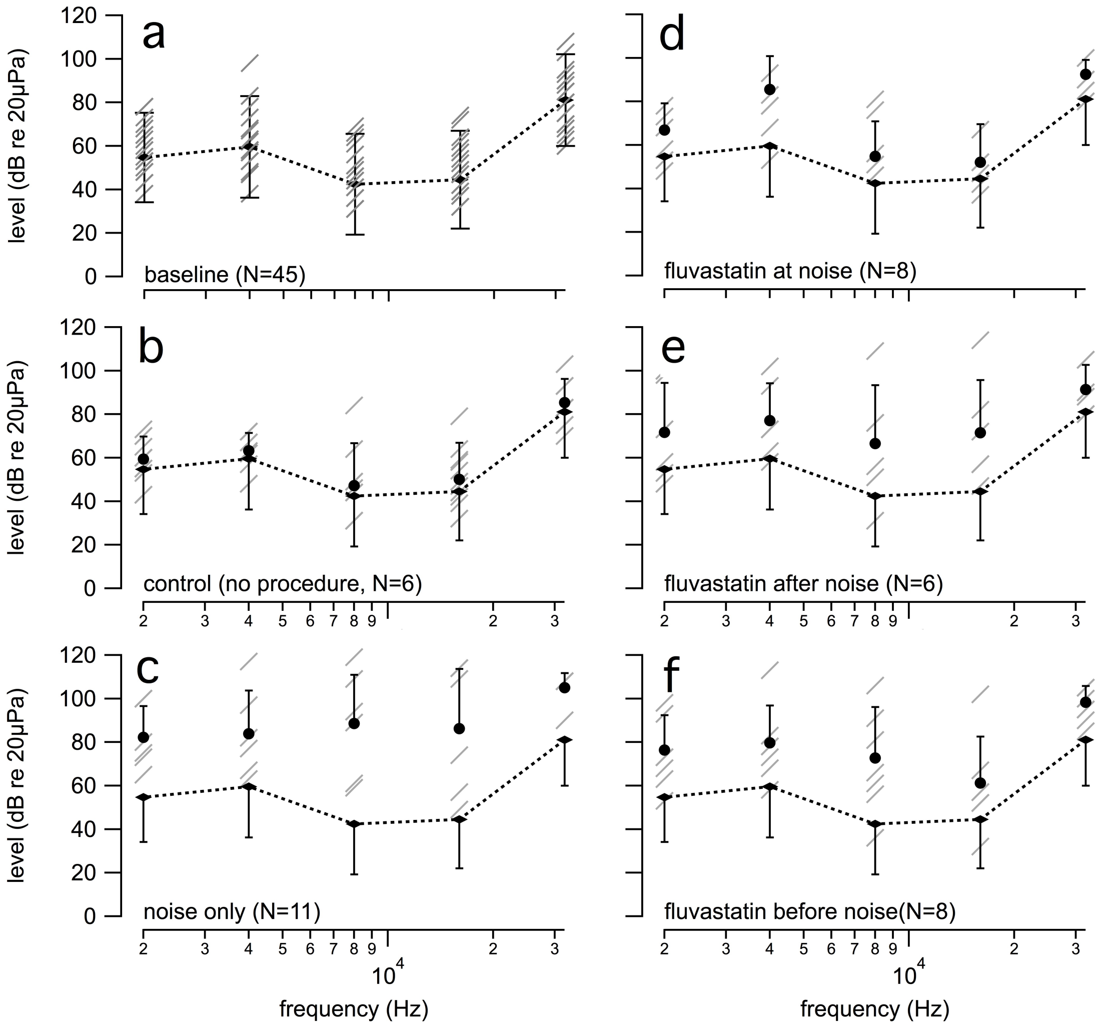

SFigure 2. ABR threshold curves

The panels show sound levels required to evoke a visible change in the recorded brainstem responses in after acoustic tone bursts. The gray lines show the individual data, the filled circles the averages ± one standard deviation. In panels (b-f), the broken line is the average of the baseline data and is shown as a reference. (a) the responses of all animals investigated before any manipulation was done to the animals. Results from left and right cochleae were pooled. (b) results from the animals of the no procedure group. Baseline threshold and end of study results are similar. (c–e) the fluvastatin treated animals and (f) the noise exposed but not fluvastatin treated animals.

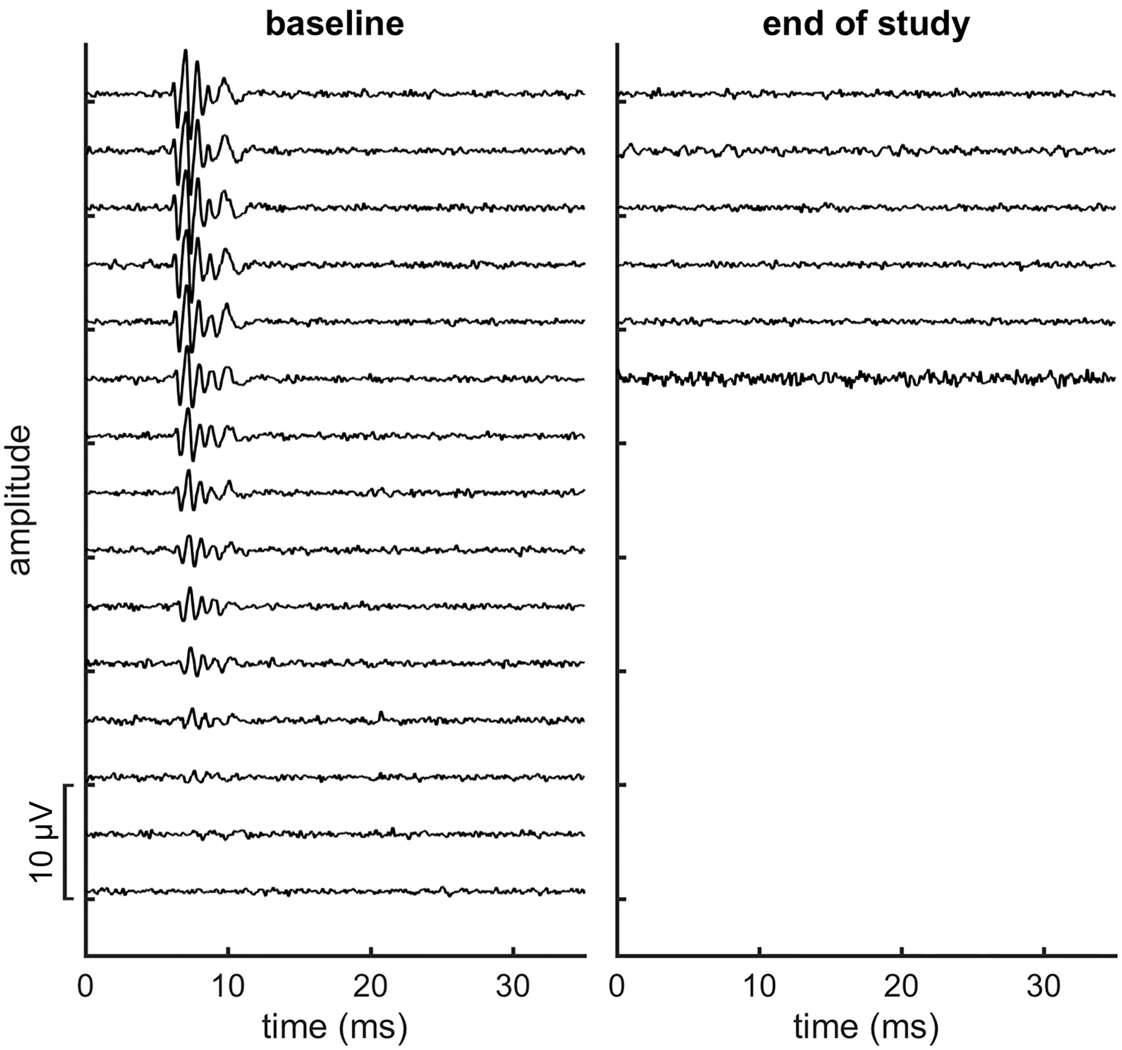

SFigure 3. ABR recordings

The plots show typical ABR responses to acoustic clicks. Baseline data are plotted in the left panel and the data at the end of the study in the right panel. The animal was treated with DMSO, no fluvastatin, and was exposed for 4 hours to 120 dB (re 20 µPa) broadband (4-8 kHz) noise. The sound level decreases from top to bottom in steps of 5 dB. The top trace has been acquired at 109.9 dB SPL peak. While the threshold in the left panel is in the second trace from the bottom (44.9 dB SPL), no ABR response could be evoked at the end of the study.

Statistics for cumulative plots of Figure 3

32kHz	nothing done	FbeforeN	FatN	FafterN	DMSO	noise only
nothing done	⊘	0.37	0.99	**0.047**	⇧	**0.0004**
FbeforeN	⊘	⊘	0.74	0.37	⇧	**0.047**
FatN	⊘	⊘	⊘	**0.047**	⇧	**0.003**
FafterN	⊘	⊘	⊘	⊘	⇧	**0.02**
DMSO	⊘	⊘	⊘	⊘	⊘	
noise only	⊘	⊘	⊘	⊘	⊘	⊘

16kHz	nothing done	FbeforeN	FatN	FafterN	DMSO	noise only
nothing done	⊘	0.39	0.97	0.22	**9.00E-04**	**2.00E-04**
FbeforeN	⊘	⊘	0.1	0.62	**0.0009**	**0.0002**
FatN	⊘	⊘	⊘	0.051	**0.003**	**0.02**
FafterN	⊘	⊘	⊘	⊘	0.1	0.051
DMSO	⊘	⊘	⊘	⊘	⊘	0.6
noise only	⊘	⊘	⊘	⊘	⊘	⊘

8kHz	nothing done	FbeforeN	FatN	FafterN	DMSO	noise only
nothing done	⊘	0.051	0.22	0.39	**7.00E-05**	**7.00E-05**
FbeforeN	⊘	⊘	0.6	0.34	0.11	0.39
FatN	⊘	⊘	⊘	0.86	**0.008**	**0.02**
FafterN	⊘	⊘	⊘	⊘	**0.021**	**0.021**
DMSO	⊘	⊘	⊘	⊘	⊘	0.86
noise only	⊘	⊘	⊘	⊘	⊘	⊘

4kHz	nothing done	FbeforeN	FatN	FafterN	DMSO	noise only
nothing done	⊘	0.86	0.62	0.39	0.08	**0.02**
FbeforeN	⊘	⊘	1	0.86	0.051	0.22
FatN	⊘	⊘	⊘	0.94	0.2	0.62
FafterN	⊘	⊘	⊘	⊘	0.39	0.6
DMSO	⊘	⊘	⊘	⊘	⊘	0.6
noise only	⊘	⊘	⊘	⊘	⊘	⊘

2kHz	nothing done	FbeforeN	FatN	FafterN	DMSO	noise only
nothing done	⊘	0.86	0.86	0.86	0.051	0.051
FbeforeN	⊘	⊘	0.86	0.86	0.39	0.39
FatN	⊘	⊘	⊘	0.86	0.051	0.51
FafterN	⊘	⊘	⊘	⊘	0.22	0.2
DMSO	⊘	⊘	⊘	⊘	⊘	0.98
noise only	⊘	⊘	⊘	⊘	⊘	⊘

STable 1. Statistics for Kolmogorov Smirnov (KS) test
To visualize the distribution of ABR responses in each group, Figure 3 shows the cumulative percent histograms of thresholds of the right ears for an auditory brainstem response (ABR) from different animals for various experimental conditions to determine whether differences in the traces are significant, the traces for each given frequency were compared using the Kolmogorov Smirnov (KS) test. It is a non-parametric test to compare the distribution of a reference and a sample. The testing was done with an $\alpha=0.05$ and $p\leq 0.05$. The experimental groups are shown along the x- and y-axis of the table. For each comparison the p-value is displayed. For the green fields the results are statistically significant. FbeforeN-Fluvastatin 1 week before noise; FatN-Fluvastatin at noise; FafterN, Fluvastatin 1 week after noise; DMSO-DMSO at noise; noise only-noise, no other treatment.

SFigure 1. Cochlear function in the implanted ear

Responses to acoustic click stimuli delivered to the left ear are shown. Average threshold elevations (average ± 1 standard deviation) for treatments with fluvastatin before, at, and after the noise exposure were 43.6 ± 14.9 dB (N=7), 25.0 ± 16.5 dB (N=8), 49.2 ± 18.8 (N=6), respectively. DMSO only treated animals had an average threshold elevation of 40.7 ± 27.1 dB (N=7). For non-treated (no surgery) but noise exposed animals the threshold elevation was 30.0 ± 21.4 (N=11); for animals without any procedure the average threshold change was 4.2 ± 4.9 dB (N=6). Differences were statistically only significant when compared with the untreated animals (DF=39, Fc=2.64, $P<0.05$). The gray circles show the corresponding data obtained in the right ear shown in Figure 2a. The threshold shifts in the left cochlea for the no procedure-unexposed and the noise only conditions were similar to those in the right ears. The other fluvastatin groups showed about a 20 dB higher threshold change in the left cochlea than in the right. The data suggest that surgery introduces a 20 dB elevation in threshold.

SFigure 2. ABR threshold curves

The panels show sound levels required to evoke a visible change in the recorded brainstem responses in after acoustic tone bursts. The gray lines show the individual data, the filled circles the averages ± one standard deviation. In panels (b-f), the broken line is the average of the baseline data and is shown as a reference. (a) the responses of all animals investigated before any manipulation was done to the animals. Results from left and right cochleae were pooled. (b) results from the animals of the no procedure group. Baseline threshold and end of study results are similar. (c–e) the fluvastatin treated animals and (f) the noise exposed but not fluvastatin treated animals.

SFigure 3. ABR recordings

The plots show typical ABR responses to acoustic clicks. Baseline data are plotted in the left panel and the data at the end of the study in the right panel. The animal was treated with DMSO, no fluvastatin, and was exposed for 4 hours to 120 dB (re 20 µPa) broadband (4-8 kHz) noise. The sound level decreases from top to bottom in steps of

5 dB. The top trace has been acquired at 109.9 dB SPL peak. While the threshold in the left panel is in the second trace from the bottom (44.9 dB SPL), no ABR response could be evoked at the end of the study.

STable 1. Statistics for Kolmogorov Smirnov (KS) test

To visualize the distribution of ABR responses in each group, Figure 3 shows the cumulative percent histograms of thresholds of the right ears for an auditory brainstem response (ABR) from different animals for various experimental conditions to determine whether differences in the traces are significant, the traces for each given frequency were compared using the Kolmogorov Smirnov (KS) test. It is a non-parametric test to compare the distribution of a reference and a sample. The testing was done with an $\alpha=0.05$ and $p\leq0.05$. The experimental groups are shown along the x- and y-axis of the table. For each comparison the p-value is displayed. For the green fields the results are statistically significant. FbeforeN-Fluvastatin 1 week before noise; FatN-Fluvastatin at noise; FafterN, Fluvastatin 1 week after noise; DMSO-DMSO at noise; noise only-noise, no other treatment.
